# Supplementary material for: Analysis of the Fungal Community in Ziziphi Spinosae Semen through High-Throughput Sequencing
Source: Toxins (Basel). 2018 Nov 25;10(12):494. doi: 10.3390/toxins10120494 (PMC6315384; doi:10.3390/toxins10120494)
Supplement: Supplementary file 1 [file toxins-10-00494-s001.pdf]

Supplementary Materials: Analysis of the Fungal Community in Ziziphi Spinosae Semen through High-Throughput Sequencing

Mengyue Guo, Wenjun Jiang, Jiaoyang Luo, Meihua Yang and Xiaohui Pang

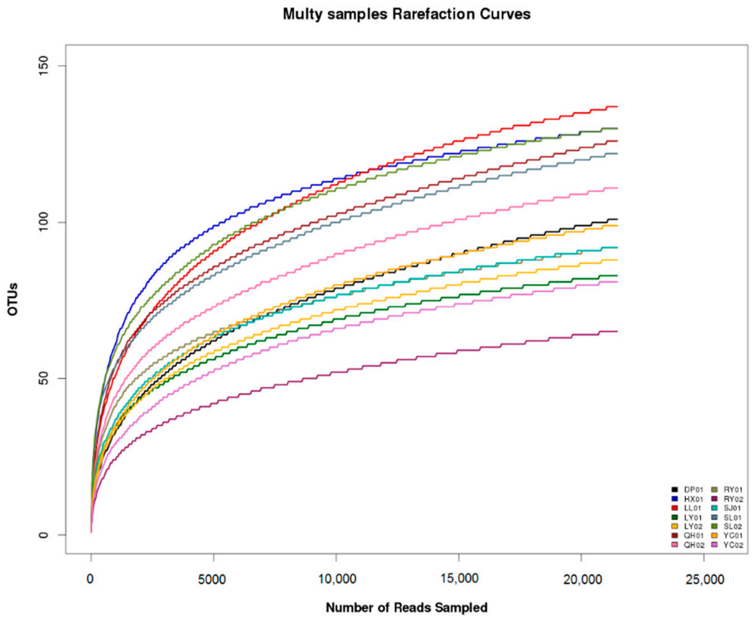

Figure S1. Rarefaction curves of OTUs for the ZSS samples.

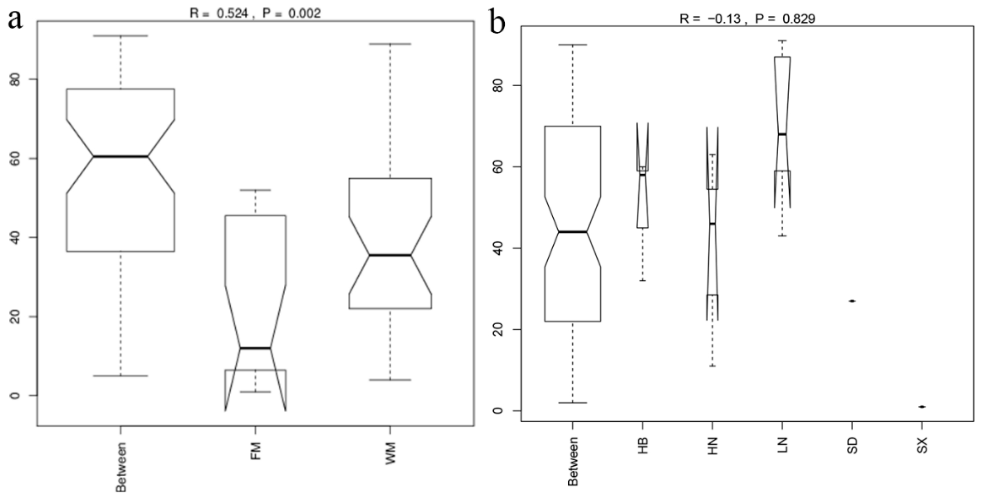

Figure S2. ANOSIM analysis of the ZSS samples. (a) The differences in the fungal community structures between the FM and WM groups. (b) The differences in the fungal community structures among samples from the five producing areas.

Table S1. α-Diversity of the fungal community in the ZSS samples.

Sample ID	Chao 1	Shannon	Good's coverage
LY01	96.68597403	2.6515269	0.999094812
LY02	109.915711	1.4940659	0.998872394
YC01	129.3203167	2.5410503	0.998562044
YC02	108.5270022	1.9618784	0.998841359
QH02	148.4468811	2.9485333	0.998355144
RY01	108.242702	2.4980974	0.998960327
RY02	76.70891414	1.8375143	0.999218952
LL01	165.0295761	3.0373105	0.998313764
HX01	147.9726282	4.223617	0.998996534
QH01	155.7804329	3.4287479	0.998479284
SJ01	114.0837485	3.0038334	0.998903429
SL01	160.5703373	3.9863995	0.998396524
SL02	158.1547728	3.9677657	0.998479284
DP01	126.6995184	3.1786958	0.998572389
